# Hybrid Fixation Achieves Similar or Slightly Better Results Compared With All Cemented Fixation in Oxford Unicompartmental Knee Arthroplasty at the Short-Term Follow-Up

**DOI:** 10.7759/cureus.81533

**Published:** 2025-03-31

**Authors:** Akira Saitoh, Takafumi Hiranaka, Koji Okamoto, Takaaki Fujishiro, Motoki Koide, Yoshihito Suda, Atsuki Tanaka, Akihiko Arimoto

**Affiliations:** 1 Orthopaedic Surgery and Joint Surgery Centre, Takatsuki General Hospital, Takatsuki, JPN; 2 Orthopaedic Surgery and Joint Surgery Centre, Takatsuki General Hospital, takatsuki, JPN

**Keywords:** cemented, complication, hybrid fixation, knee osteoarthritis (koa), unicompartmental knee arthroplasty (uka)

## Abstract

Introduction

Cementless Oxford unicompartmental knee arthroplasty (OUKA) has been shown to have less frequent radiolucent lines and equivalent or even better results than those of cemented OUKA. However, tibial fractures are more frequent in cementless OUKA than in cemented OUKA, especially in Asian countries. A hybrid option, with a cementless femur and cemented tibia, may, therefore, be a good compromise. This study compares the clinical results of hybrid OUKA with those of fully cemented OUKA.

Materials and methods

This retrospective study included 108 consecutive unicompartmental knee arthroplasties implanted between September 2016 and September 2018 in our hospital. Cases were divided into two groups: those using cemented fixation and those using hybrid fixation OUKAs. Pre- and postoperative knee range of motion (ROM), operation time, pre-and postoperative Oxford knee score (OKS), and complications were compared between the groups two years after OUKA was performed.

Results

There was no significant difference in pre- and postoperative ROM, operation time, and OKS performed unilateral OUKA, but mean OKS was higher in the hybrid group than in the cemented group performed bilateral OUKA (p<0.01). Both groups included one revision to total knee arthroplasty each. There was no statistically significant difference in the rate of revision (p=0.723).

Conclusions

Better clinical outcomes were achieved in the hybrid fixation group than in the cemented fixation group, with an equivalent rate of complication. Longer follow-up periods are needed to confirm the benefits of hybrid fixation in OUKA over time.

## Introduction

Oxford unicompartmental knee arthroplasty (OUKA) has been reported to achieve better clinical results than total knee arthroplasty (TKA). The advantages of unicompartmental knee arthroplasty (UKA) are reduced invasiveness, faster recovery, and fewer systemic complications and mortality compared with TKA [1-4]. Price et al. [5] reported that the 20-year cumulative survival rate after UKA was 91%, and Lisowski et al. [6] reported a 15-year survival rate of 90.5%. In contrast, most national joint registries have reported higher rates of revision in UKA than in TKA, with the most common causes of the need for revision being aseptic loosening and unexplained medial-sided pain [3].

One reason for such a high revision rate is the misinterpretation of the radiolucent lines (RLLs) at the bone-component interface as an aseptic loosening; this could lead to unnecessary revision [7,8]. Cementless OUKA has been used since 2004. Radiolucency around the components is rare in cementless OUKA compared with cemented OUKA [9,10]. Moreover, equivalent or even better results have been reported compared with cemented OUKA [11]. Furthermore, no time is needed for cement preparation, and there are no cement-related complications, such as cement fragment impingement [12]. In contrast to reports from Western countries, tibial fractures are more commonly reported in Asian countries after cementless OUKA, perhaps owing to the smaller bone size and overhanging medial tibial condyle due to the proximal tibia vara [13]. To avoid serious complications, a hybrid fixation option, using a cemented tibia but with a cementless femur, is suggested to be a reasonable option because of the reduction in tibial fracture risk, avoidance of cement-related trouble, and acquisition of enough cementing time for tibial fixation. Despite the potential advantages however, hybrid fixation Oxford UKA has few reports in detail [14]. This study, therefore, compares the clinical results between hybrid and fully cemented fixation in OUKA. Hybrid OUKA fixation may be able to obtain equivalent clinical outcomes and complications to fully cemented OUKA but with the aforementioned benefits.

## Materials and methods

This retrospective longitudinal study was approved by the appropriate institutional review board, and written informed consent was obtained for each patient. This study includes 108 consecutive UKAs implanted between September 2016 and September 2018 in one hospital. Cementless OUKA became available in Japan in September 2015, and until then, cemented OUKA had been performed in all cases. Since cementless OUKA became available, it has been used in most cases. Hybrid OUKA, in which only the tibia is cement-fixed, was only performed for patients who had their tibia recut during surgery or who had poor fixation during a tibia trial. Due to the frequent occurrence of postoperative tibial plateau fractures, however, we switched to hybrid OUKA in all cases. The hybrid group comprised 57 knees in 41 patients who underwent hybrid OUKA (nine males, 32 females, 56-85 years of age) during the study period. The cemented group comprised 51 knees in 36 patients that underwent cemented OUKA (eight males, 28 females, 58-91 years of age). All patients were followed up at least two years postoperatively. There were no statistically significant differences in the demographic data (Table 1).

**Table 1:** Patient demographics OA - osteoarthritis; SONK - spontaneous osteonecrosis of the knee

Variables	Cemented group ( n = 51)	Hybrid group ( n = 57)	p-value
Age (years)	75.1	73.3	0.348
Gender (male:female)	8:28	9:32	0.596
OA:SONK	40:11	51:6	0.185

All patients underwent UKAs with mobile-bearing OUKA (Zimmer Biomet, Warsaw, IN) and had anteromedial osteoarthritis (OA) or spontaneous osteonecrosis of the knee (SONK) and conformed to the indications based on radiographical decision aid [15]. Patients had medial osteoarthritis, correctable varus deformity, an intact anterior cruciate ligament, and collateral ligaments and there were no degenerative findings in the lateral compartment of the knee on standing radiographs and valgus stress radiographs. Surgery was performed by the senior author, who is experienced in both TKA and OUKA, or under his direct supervision. The under vastus approach and the microplasty instruments were used in accordance with the manufacturer's instructions [16,17]. In the case of bilateral OUKA, anesthesia was administered once with general anesthesia. The surgery was performed by a single team. Draping was done on both sides at the same time, and as soon as one side was finished, surgery on the other side began immediately.

Pre- and postoperative knee range of motion (ROM), operation time, pre-and postoperative Oxford Knee Score (OKS), RLLs, and complications were compared between the groups. Regarding the ROM, operation time, and OKS, bilateral operation cases and unilateral operation cases were compared, respectively.

**Figure 1:**
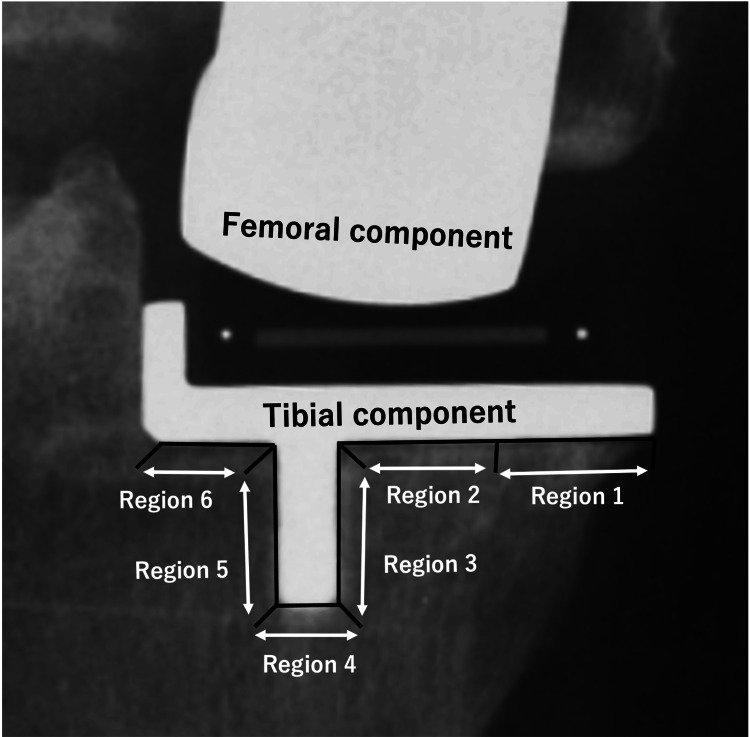
Radiograph coronal view of tibial component Radiograph showing the six regions for the distribution of radiolucent lines beneath the tibial component of the Oxford UKA UKA - unicompartmental knee arthroplasty

**Figure 2:**
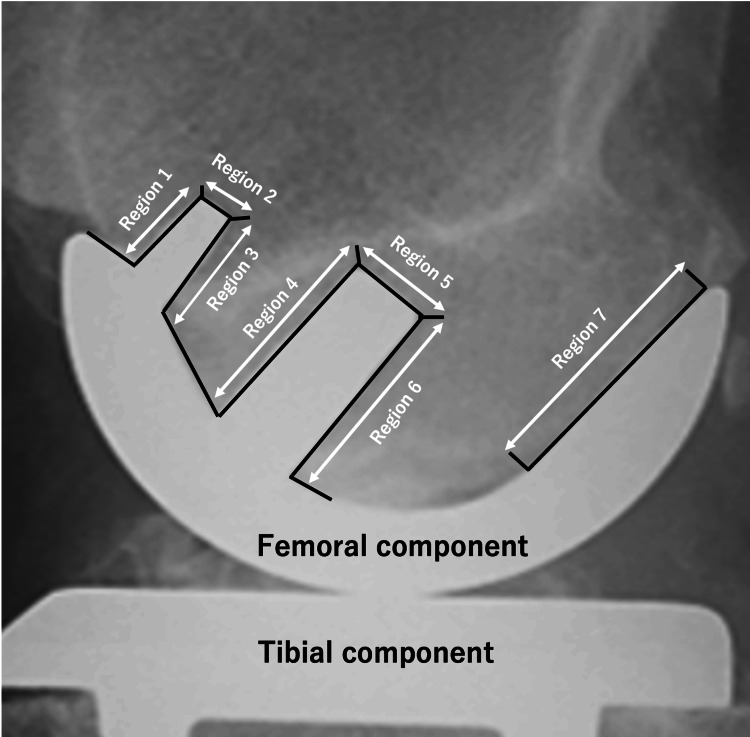
Radiograph sagiital views of femoral component Radiograph showing the seven regions for the distribution of radiolucent lines beneath the femoral component of the Oxford UKA UKA - unicompartmental knee arthroplasty

Evaluation of RLLs was performed by anteroposterior radiography for the tibial side and lateral view for the femoral side. The area under the tibial tray was divided into six areas, and the femoral side was divided into seven (Figures 1, 2) [18]. RLL was defined as a progressive radiolucency around the complete component >1 mm thick with no adjacent sclerosis [19].

In each area, the presence or absence of the RLL was recorded. Their distribution in each patient was classified into three types: all regions had RLL (complete), no regions had RLL (none), and others (partial) [19]. The occurrence of complications and revision was compared between the groups.

The student's t-test was used to compare the difference in ROM and OKS, and Fisher's exact test was performed to compare the rate of complications and revisions between the groups. All statistical analyses were performed using EZR (easy R) running on R, and all measurements are expressed as mean ± standard deviation [20].

## Results

Clinical outcomes are shown in Table 2 and Table 3.

**Table 2:** Clinical outcomes of bilateral OUKA OUKA - Oxford unicompartmental knee arthroplasty; ROM - range of motion; OKS - Oxford Knee Score

Bilateral OUKA	Cemented group (n=15)	Hybrid group (n=16)	p-value	t value
ROM (flexion, degree)	131.2 ± 13.1	135.5 ± 15.4	0.266	-1.163
ROM (extension, degree)	-0.3 ± 1.2	-0.9 ± 2.6	0.249	1.124
OKS	33.8 ± 8.3	40.8 ± 4.2	<0.01	-2.864
Operation time (min)	88.5 ± 17.4	89.5 ± 18.0	0.869	-0.176

**Table 3:** Clinical outcomes of unilateral OUKA OUKA - Oxford unicompartmental knee arthroplasty; ROM - range of motion; OKS - Oxford Knee Score

Unilateral OUKA	Cemented group (n=21)	Hybrid group (n=25)	p-value	t value
ROM (flexion, degree)	138.8 ± 19.5	141.2 ± 9.7	0.600	-0.528
ROM (extension, degree)	-1.0 ± 3.3	-0.4 ± 1.4	0.461	-0.744
OKS	36.9 ± 8.4	38.8 ± 6.4	0.410	-0.831
Operation time (min)	65.7 ± 14.1	63.1 ± 13.7	0.534	0.627

There were no significant differences in the pre- and postoperative ROM and operation time, but mean OKS was higher in the hybrid group than in the cemented group at bilateral OUKA (p<0.01).

The radiolucent lines of the tibial component were found in 17 cases (partial: 12, complete: 5) in the cemented group and 15 cases (partial: 5, complete: 10) in the hybrid group (p=0.074). Regarding the femoral side, there was no obvious radiolucent line in either group. There were ten complications in the cemented group and four complications in the hybrid group (Table 4).

**Table 4:** Complications and reasons for revision

Complication	Cemented group (n=10)	Hybrid group (n=4)	p-value
Tibial plateau fracture	3	0	0.505
Subsidence of tibia component	4	2	1.000
Hemarthrosis	2	1	1.000
Wound problem	1	1	0.506
Reasons for revision	Tibial plateau fracture	Subsidence of tibia component	
Revision Rate (%)	1.96	1.75	0.723

There was no significant difference in complications. Tibial plateau fracture occurred in three patients in the cemented group and no patients in the hybrid group. One patient was converted to TKA, and the other two patients required open reduction and fixation. Subsidence of the tibial component occurred in four patients in the cemented group and two patients in the hybrid group. One patient in the hybrid group had a fall after the subsidence of the tibial component and was converted to TKA. The other patients were treated conservatively. Hemarthrosis occurred in two patients in the cemented group and one patient in the hybrid group. There was delayed wound healing in one patient in each of the groups. The revision rate was 1.96% (one knee) in the cemented group and 1.75% (one knee) in the hybrid group, without statistical significance (p=0.723). No complications related to the femoral component were found in either group.

## Discussion

Better clinical outcomes were acquired in the hybrid fixation group than in the fully cemented fixation group in this study. Previous studies reported that the clinical outcome was similar or even superior in cementless OUKA compared with the cemented OUKA [21,22]. Cementless OUKA has a potential advantage regarding less frequent radiolucency and no cement-related trouble, such as residual cement fragments [11,23]. Hybrid OUKA can, therefore, acquire superior results to cemented OUKA. Although a historical comparison was performed in this study, leading to bias, this is the first detailed examination of the hybrid option of OUKA, and the results are encouraging.

Operation time did not differ between the two groups studied. Although the operation manual provided by the manufacturer recommends the two-cement technique, where femoral and tibial cementing are performed separately using two sets of cement [2], we always use the one-cement technique, where both tibial and femoral cementing are done with one cement set. The femoral side procedure can be completed during one cementing session, so the operation time is similar. However, it might be beneficial for surgeons to have enough time spare for tibial cementing if it becomes necessary. The insertion time in hybrid fixation is equivalent to the cemented fixation. Surgeons may benefit from more time devoted to tibial preparation by not having to fix the femur with cement.

Our results also showed no differences in the occurrence of RLL. The cementless fixation of the femur had no particular effect on the occurrence of RLLs. The occurrence of the RLL in the femur is less frequent than in the tibial side in cemented OUKA. Similarly, RLL has been shown in previous studies to be infrequent in cementless OUKA compared with cemented OUKA [24,25]. Our results are thus compatible with the previous reports; RLL occurrence was similar in both groups, which is understandable because cemented tibia were used in both groups. The cementless femur does not, therefore, increase the occurrence of RLL and is thus recommended.

**Figure 3:**
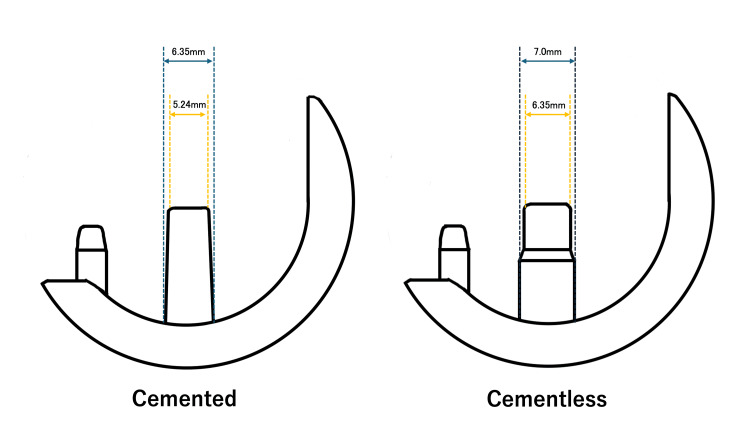
A sagittal view of Oxford femoral components A sagittal view of Oxford femoral components comparing the size and shape of pegs with cement and cementless

The complication and revision rates were similar between the groups, and all implant-related failure occurred in the tibial side, there was no failure in the femoral component. Primary fixation was enhanced in the cementless femoral component by the press-fitting of the main peg with 0.65 mm of interference (the peg of 7.00 mm in diameter into the drill hole of 6.35 mm in diameter; Figure 3).

Moreover, the posterior condyle is cut in 1.5° extension position with respect to the peg holes for a secured contact between the bone and component. This is considered to be the reason why there are few complications on the femur side. Inui et al. [26] reported two cases of loosening of the femoral cementless component, but there are no other reports of complications around the femoral component of cementless OUKA until now. The probability of complications around the femoral component of cementless OUKA is, therefore, suggested to be extremely low. The hybrid option benefits from secure and safe tibial fixation and simple and cement-related, trouble-free fixation of the femur.

There are several limitations to this study. Firstly, it is retrospective and not a randomized controlled trial, and a historical comparison was performed. However, the operative procedure and patient selection was the same in both groups. Secondly, the anteroposterior radiography for the tibial side was not taken under fluoroscopy, so there may be an underestimation of RLLs. Finally, the follow-up period was about two years - a longer follow-up with a larger number of patients is preferable. Despite the limitations, the study suggests that the performance of hybrid OUKA infers a possible decreased risk of cement-related problems and better clinical outcomes compared with cemented OUKA.

## Conclusions

Cemented OUKA and hybrid OUKA were not significantly different in terms of postoperative ROM, operation time, and the number of complications. There were no complications on the femoral side in either group. To avoid cement-related complications, hybrid UKA with cementless fixation on the femoral side and cemented fixation on the tibial side is of interest. Hybrid OUKA had improved OKS, but longer follow-up periods are needed to confirm whether it should be chosen preferentially. A comparative study is also needed regarding cemented, hybrid, and cementless fixation.
